# Advancing Spectroscopy‐Based Drug Checking Training: A Proof‐of‐Concept Study for the Use of Concordance Analysis

**DOI:** 10.1111/dar.70246

**Published:** 2026-09-06

**Authors:** Yarelix Estrada, Jeffery Sauer, Leonardo Dominguez, Mia Borrelli, Sarah A. Shuda, Amanda L. A. Mohr

**Affiliations:** ^1^ New York City Department of Health and Mental Hygiene Bureau of Alcohol and Drug Use New York New York USA; ^2^ Center for Forensic Science Research and Education at the Fredric Rieders Family Foundation Horsham Pennsylvania USA

**Keywords:** accuracy assessment, concordance analysis, drug checking, FTIR, GC/MS, LC‐QTOF/MS

## Abstract

**Introduction:**

Drug checking is a harm reduction strategy that provides information to people who use drugs. Currently, there is a lack of standardised, replicable methods to assess drug checking technicians' use of spectroscopy‐based technologies.

**Methods:**

This paper introduces concordance analysis as a method to assess drug checking technician accuracy using Fourier transform infrared (FTIR) spectroscopy. Drug checking technicians provided point‐of‐care drug checking services, including FTIR, at participating syringe service programs in New York City beginning in November 2021. Drug checking technicians analysed drug samples and identified compounds based on spectra, test strips and contextual information provided by the individuals submitting samples. The drug checking technician results were compared to results obtained from a secondary laboratory testing partner. Technicians aimed to achieve a 90% concordance rate for sample composition determinations in the field as compared to secondary laboratory testing results.

**Results:**

Two drug checking technicians encountered a total of *N* = 362 samples over the course of their training periods. Concordance analysis showed that both technicians completed their training period above the target accuracy level (Technician A 137/151 or 90.7% concordant, Technician B 196/211 or 92.9% concordant). The training periods for Technician A and Technician B were 33 and 38 weeks, respectively. Both technicians demonstrated improvement over time.

**Discussion and Conclusions:**

These results indicate that drug checking technicians with no formal background in analytical chemistry are able to accurately identify compounds present in real‐world drug samples using an FTIR, thus supporting the merit of these techniques in drug checking programs.

## 1 Introduction

Drug checking is a harm reduction strategy that seeks to provide people who use drugs (PWUD) with actionable information as well as offer relevant organisations with insight into the unregulated drug supply. Drug checking services have been established across Europe for several decades [1] and, more recently, drug checking services have rapidly expanded across North America [2]. Within the last few years, government agencies have begun allocating resources and technical assistance to expand drug checking across jurisdictions in the United States as one of the many responses to the overdose epidemic.

Drug checking technologies exist in many forms, with each technology having nuanced limitations. Common drug checking technologies include immunoassay test strips, colorimetric reagent tests and spectroscopy [3]. Test strips provide a binary indication as to the presence of a compound and, depending on the manufacturer and target compound of interest, can detect small amounts of a substance [4]. While the use of immunoassay test strips has been associated with a variety of individual‐level harm reduction behaviours, the strips themselves have also been shown to exhibit batch‐by‐batch variability and cross‐reactivity with other substances [5]. In addition, drug samples may contain multiple compounds at different concentrations, which motivates the use of other drug checking technologies. Spectroscopy‐based technologies—such as attenuated total reflectance‐infrared (ATR‐IR) spectroscopy, Raman spectroscopy, Fourier transform infrared (FTIR) spectroscopy, gas chromatography/mass spectrometry (GC/MS), among others—vary in cost, required training for successful operation and suitability for use in the field, though they all have the ability to complement other field‐ready technologies like immunoassay test strips. In particular, spectroscopy‐based technologies can identify multiple substances and, under certain conditions and with the appropriate training, can be used to approximate the concentration of compounds present in a sample or produce the data required for a statistical model that approximates the compound concentration [6]. A recent review of drug checking services highlighted the benefit of multi‐instrument drug checking approaches, namely due to the fact that multiple technologies, when used in combination, can overcome technology‐specific limitations and provide more information to people using the service [7].

While spectroscopy‐based technologies are increasingly prevalent as an offering of point‐of‐care drug checking programs, the quality of information derived from these technologies is subject to a variety of complicating factors. For example, in the case of infrared spectroscopy, complicating factors can include heterogeneity of samples, the amount of sample placed on the FTIR and its contact with the crystal, the concentration and number of components present that absorb infrared radiation, the spectral similarity of components and, more simply, whether a compound is present in reference libraries available on a given spectroscopy software platform [3]. Recent work has demonstrated that data produced from spectroscopy‐based instruments can be used to train a model that automatically classifies compounds present in a sample [8] and such efforts would likely benefit from large, open datasets built from data representing drug samples acquired from community settings. However, access to and the use of such automated tools remain limited. As such, manual operation by trained drug checking technicians remains the predominant form of providing spectroscopy‐based drug checking services. In the absence of other improvements—such as instrument limits of detection and techniques to differentiate related compounds—the previously mentioned complicating factors will continue to impact the ability of drug checking technicians to provide accurate information to people using drug checking services. Ensuring high‐quality training and ongoing quality assessment of drug checking technicians is essential, especially in the context of an unpredictable and continually evolving drug supply and as drug checking programs continue to expand.

Previous studies have generally focused on demonstrating that spectroscopy‐based technologies can identify compounds of interest in drug samples acquired from community settings [9, 10, 11]. For example, a study from Green et al. calculated the sensitivity and specificity of three technologies—fentanyl immunoassay test strips, Raman spectroscopy and FTIR—in their ability to detect fentanyl compared to known results from GC/MS for drug samples obtained in Baltimore, Maryland and Providence, Rhode Island [10]. Another study by Laing et al. calculated false positive and negative rates for fentanyl and benzodiazepines using test strips and FTIR, again in comparison to GC/MS [12]. While these studies and others are useful in understanding the limits and nuances of specific drug checking technologies in identifying specific compounds, drug samples are often comprised of multiple compounds, each of which may be active and play a role in the health outcomes of a person who uses drugs [13]. Thus, in addition to ensuring that the technologies themselves can identify specific compounds, attention must also be paid to the accuracy of the technician operating a spectrometer. Given the complexity of drug samples from community settings, especially those that check opioid samples, technician accuracy post‐training should reflect their ability to identify as many of the compounds present in the sample that are detectable given technological limitations.

To the best of the authors' knowledge, there exists no generally accepted proficiency testing methodology or accuracy standards for the practice of FTIR‐based drug checking in the field. While organisations providing drug checking services have begun publishing detailed program implementation guides [14, 15], information on technician training protocols and subsequent evaluations is typically only available internally to each program. However, related fields, such as analytical chemistry, have previously grappled with similar issues and have produced a set of generally accepted scientific concepts and practices from which drug checking can draw. For example, several organisations providing services involving analytical chemistry, such as forensic toxicology laboratories, use accreditation requirements and standard practices for proficiency testing set by recognised professional organisations [16, 17]. Of relevance to drug checking is the idea of advancing a set of procedures, proficiency testing methods and accuracy standards when using spectrometry‐based technologies for drug checking in real‐world settings. Working to advance a set of generally accepted standards can help further the legitimacy of drug checking as a rigorous, evidence‐based service that can provide PWUD with high quality, actionable information.

The objective of this paper is twofold. First, we describe a practical methodology for assessing drug checking technician accuracy called concordance analysis. Second, we provide data on concordance analysis for two drug checking technicians as evidence of proof of concept in a real‐world setting. The paper includes details on the concordance analysis used during a drug checking pilot study launched in a major metropolitan area of the United States, the secondary laboratory testing procedures that provided the reference results for evaluating technician accuracy and examples of complicated scenarios encountered in concordance analysis. In doing so, we aim to advance the standards of drug checking technician training, with the goal of providing accurate information to PWUD accessing point‐of‐care drug checking services using these technologies.

## 2 Methods

The following section describes the key context and details underpinning different facets of the concordance analysis. The section begins with an explanation as to the public health problem and drug checking technologies featured in the concordance analysis (Section 2.1). The section continues with details on the secondary laboratory testing laboratory procedures used to generate comparison for results obtained through point‐of‐care drug checking (Section 2.2). The section concludes with an explanation as to how the concordance analysis was conducted and the results of technician concordance over time (Section 2.3).

### 2.1 A Point‐of‐Care Drug Checking Pilot Study in New York City

Beginning in November 2021 and continuing through December 2023, the New York City Health Department partnered with community‐based syringe service programs (SSP) to conduct a drug checking pilot study. This study offered multiple drug checking technologies including immunoassay test strips, FTIR spectroscopy and the option for additional secondary laboratory testing at existing sites where PWUD can access other harm reduction, therapeutic and medical services. Drug checking technicians recruited SSP participants into the pilot study via intercept with the programs, advertised the study within the SSP and received referrals via SSP staff. Drug samples of any class could be submitted to the pilot study. The study eligibility criteria included those SSP participants over the age of 18 who currently used drugs and spoke English or Spanish.

All New York City Health Department drug checking technicians underwent pre‐implementation training before conducting the point‐of‐care drug checking offered in the drug checking pilot study. The training included a review of drug checking literature and tutorials from existing programs, more than 24 h of direct training from an experienced drug checking technician, supervised analysis of existing data with known results and shadowing an experienced drug checking technician at an active point‐of‐care drug checking service.

Once the technician completed pre‐implementation training, they began providing drug checking for the pilot study in their training phase. During the training phase, each technician provided drug checking services 1 day a week during the regular operating hours of a participating site. Each sample submitted for drug checking was sent for secondary laboratory testing. This allowed the technician to compare their list of compounds identified to an external, independently generated list of compounds provided by a secondary laboratory testing partner with more sophisticated analytical methods. All participants who used the drug checking service during a technician's training phase were informed that the technician was in training, received a disclaimer explaining the limitations of the technician being in training as well as the limitations of the technology and were compensated with a $25 gift card. Each participant could receive one gift card per day and gift cards were no longer distributed once a technician was considered trained (see Section 2.3 for a detailed description of concordance analysis and the benchmark set by the program). Technicians used all information available to them—including information provided by the participant, test strip results and FTIR results—to create a list of a final set of compounds thought to be present within each sample. All test strips were procured from the medical supplier BTNX.

Each drug sample was assigned a unique sample identification code. Technicians recorded drug sample characteristics and point‐of‐care drug checking results for each uniquely identified sample using a digital survey hosted via REDCap software. Portions of each sample sent for secondary laboratory testing were labelled with the unique identifier. Survey results were then matched to secondary laboratory results using the unique sample identification code. Given the focus of the present analysis on technician accuracy assessment, we encourage readers interested in an additional description of the drug checking program to refer to details published elsewhere [15, 18].

#### 2.1.1 Description of Drug Checking Technician FTIR Operation

Once entered into the study, SSP participants provided a drug sample to the technician for analysis. A Bruker Alpha II FTIR spectrometer was used as the spectroscopy‐based technology offered in the point‐of‐care pilot study alongside a computer running the Bruker OPUS Drug ID Wizard Software. Upon setting up the instrument, the technicians cleaned the plate with three alcohol prep pads and dried the plate with a KimTech Science Kimwipe. The drug checking technicians then ran a preliminary OPUS performance qualification to ensure proper instrument functionality. The drug checking technicians then measured the background signal using built‐in background monitoring tools available in the OPUS Drug ID Wizard software to ensure that the instrument was prepared to check a new sample. The background monitoring check may fail if the plate or crystal was not properly cleaned or if the atmospheric conditions are not optimal for a new scan. The instrument's software notifies the user if a background monitoring check passed or failed. Once the background monitoring check cleared, the technician then loaded the sample by placing a small amount of the drug sample onto the FTIR crystal using sterilised stainless‐steel tools. A small piece of aluminium foil was placed on top of the sample and the FTIR anvil was secured into place to produce the scan. Technicians then measured the sample, which resulted in a preliminary spectrum of the substance. The spectrum was evaluated using both vector normalisation and first derivative transformation. Possible compound matches to the spectra were limited to the 100 top results with a hit quality above 50.

The following compound libraries were available to the technicians: (i) TICTAC spectral library [19]; (ii) British Columbia Center for Substance Use FTIR‐ATR spectral library [20]; (iii) Scientific Working Group for the Analysis of Seized Drugs spectral library [21]; and (iv) proprietary Bruker Pharma libraries. Technicians would often turn different libraries on or off as the reference scans present in the libraries, as well as the combination of different libraries, could lead to different reference spectra that could be used for analysis. Technicians examined the scan in relation to the list of compounds identified by the software to curate a list of possible substances in the sample, the results of the immunoassay test strips used for the sample, what the sample was sold as, what substances are typically found in each drug class in the local drug supply, participant experience and other pieces of information obtained by the technician. Technicians also auto‐subtracted signals for compounds thought to be present in the sample. An upper limit of identifying six compounds in a sample was established.

Once FTIR interpretation was completed, the technician recorded the FTIR results in a digital survey as well as their personal notebook. The scan was saved to the computer using an anonymous reference number for follow‐up examination, if needed. The anvil from the FTIR instrument was lifted and the aluminium foil removed, allowing the remaining sample to be tested with immunoassay test strips and sent for secondary laboratory testing to the external partner laboratory. The piece of foil was immediately discarded. The FTIR plate and anvil were then cleaned with three alcohol wipes and dried with a KimTech Science Kimwipe. After cleaning, a background scan was taken to ensure the instrument was ready for the next drug sample.

### 2.2 Description of Secondary Laboratory Testing Procedures

Drug checking samples were submitted to the Center for Forensic Science Research and Education (CFSRE) in Horsham, Pennsylvania. CFSRE was contracted to provide external, independent chemical analysis of drug samples obtained by the New York City Health Department Drug Checking Program. Samples were analysed by both GC/MS and liquid chromatography (LC) quadrupole time of flight mass spectrometry (LC/QTOF‐MS). Commercially available library databases from National Institute of Standards and Technology, Cayman Chemical and the Scientific Working Group for the Analysis of Seized Drugs were used along with a custom in‐house library for GC/MS data processing. For LC/QTOF‐MS, a custom database maintained by CFSRE was used, which includes more than 1200 analytes. In‐house libraries for both GC/MS and LC/QTOF‐MS were built using standard reference material and internally verified prior to being used in analysis. Analytes were identified based on previously established performance criteria. For GC/MS, analytes were required to have a signal to noise ratio greater than or equal to four, acceptable chromatographic peak shape, retention time ± 0.1 min of the standard, match factor of 60 or above compared to the library and visual similarity to the spectrum of the standard. For LC/QTOF‐MS, analytes were required to have a signal to noise ratio greater than or equal to 10, acceptable chromatographic peak shape, retention time within ±0.25 min of the standard in the database, < 5 ppm (ppm) mass error, peak area > 800, < 30 isotope ratio, library score greater than 90 compared and visual similarity to the TOF parent and fragment mass spectrums. Samples were considered positive when a compound was identified on both GC/MS and LC/QTOF‐MS according to internal quality assurance guidelines maintained by CFSRE. We use the term ‘secondary laboratory testing’ to refer to the results of the chemical analysis and associated services provided by CFSRE.

#### 2.2.1 Sample Preparation for Secondary Laboratory Testing

Samples received consisted of powdered material or residues. Powdered material was sampled into a test tube for extraction. Residues were sampled by swabbing the paraphernalia or bag submitted with a cotton‐tipped applicator wetted with methanol. The swab was then placed into a test tube with 1 mL of methanol and agitated to dissolve the sample into the methanol. The swab was discarded and the methanol was subjected to the extraction procedure.

Samples were prepared using an acid–base liquid/liquid extraction. Analytes were portioned between water and 90:10 dichloromethane/isopropanol. Samples were acidified using 10% hydrochloric acid and basified with concentrated ammonium hydroxide. The acidic and basic fractions were combined for analysis. The extract was transferred to an autosampler vial for GC/MS analysis. The extract was further diluted 1:50 in mobile phase for analysis by LC/QTOF‐MS.

#### 2.2.2 Instrumental Analysis

GC/MS analysis was performed on an Agilent 7890 GC coupled to a 5975 MSD using a J&W DB‐1 12 m × 0.2 mm × 0.33 μm column. The injection was performed in splitless mode with a 1 μL injection at 265°C. The mass spectrometer was operated in scan mode from 550 to 40 m/z with a source temperature of 230°C.

LC/QTOF‐MS analysis was completed using a SCIEX TripleTOF 5600+ quadrupole time‐of‐flight mass spectrometer coupled with a Shimadzu Nexera XR ultra‐high performance LC. Chromatographic separation was achieved using a Phenomenex Kinetex C18 analytical column (3.0 mm × 50 mm × 2.6 μm) using gradient elution. Column temperature was 30°C, flow rate was 0.4 mL/min and injection volume was 5 μL. Positive electrospray ionisation was used and the final run time was 15.5 min.

### 2.3 Concordance Analysis

#### 2.3.1 Concordance Analysis in Practice

Concordance analysis involves directly comparing technician composition determination in the field (i.e., using contextual information, test strips and FTIR) and secondary laboratory testing (i.e., GC/MS and LC/QTOF‐MS) results for the active substances present in each drug checking sample. Personnel involved in the concordance analysis included a technician‐in‐training and a supervising trained technician. During the training period, the technician in training manually collates the two sets of results (i.e., compounds identified by the drug checking technician versus the compounds identified by the secondary laboratory testing) to provide initial determinations as to whether their analysis was ‘Concordant’ or ‘Discordant’. All initial concordance determinations were reviewed by the supervising trained technician. This encouraged technicians in training to follow along in the progression of their training and note any recurring issues. A supervising trained technician was identified using community‐based competencies including someone who had previously reached concordance, demonstrated aptitude and capacity for training others and had experience managing drug checking programs; as of this writing there remains no centralised accrediting body for certifying drug checking trainers.

A sample was considered ‘Concordant’ based on multiple decision criteria. Compounds observed by the technician via FTIR were compared to the compounds observed via secondary laboratory testing, excluding inactive substances, related compounds, precursors, metabolites or substances found in trace amounts by the secondary laboratory in the sample. If a technician identified all active substances, the sample was marked concordant. Discretion was permitted by the supervising trained technician to account for practical considerations like instrument limits of detection, quality and availability of compounds within FTIR reference libraries, known instances of spectral overlap in the OPUS Drug ID Wizard among commonly observed compounds and trainee knowledge at that point in time. For example, a sample could still be considered concordant if a technician missed components that were at or under the approximate 5% FTIR detection limit or had significant overlap with another signal (e.g., caffeine masking the presence of fentanyl).

A sample could be considered ‘Discordant’ due to multiple reasons. If the technician missed an active substance that was reported in the secondary laboratory testing results or identified an additional active substance via FTIR that was not present in the secondary laboratory testing results, the sample was marked as discordant. The latter reason for discordance was particularly important to flag during the training process to avoid practices where technicians identified substances that would not make sense within the context of the drug class or the local drug supply. Concordance determinations for samples where missed compounds may have been below the detection limit or masked by another compound were made by a supervising trained technician, with additional contextual input by the secondary laboratory testing partner when needed.

In a few instances, drug checking technicians observed compounds via FTIR that did not meet the in‐house observability criteria of the secondary laboratory testing partner. In these instances, a sample could be considered concordant if there were any signs that the compound could be present in their analysis. For example, compounds could be detected by LC/QTOF‐MS alone due to the assay's lower limit of detection in comparison to the GC/MS. Resolving these cases required close coordination between the technician in training, technician trainer and secondary laboratory staff.

Concordance analysis was carried out separately for Technician A and Technician B of the New York City Department of Health and Mental Hygiene drug‐checking program. A technician's concordance rate was updated each time new secondary laboratory results became available. Concordance analysis included all samples for which FTIR and secondary laboratory testing results were available. Cumulative concordance rate was calculated as the number of concordant samples divided by the total number of samples tested by each technician across the entire training period. Concordance was also visualised over time to examine patterns in technician accuracy as their training periods progressed.

#### 2.3.2 Selection of an Accuracy Benchmark

The New York City Health Department set a target measure of a cumulative 90% concordance rate by the end of each technician's training period. The measure was selected in the absence of systematic studies of drug technician training proficiency and in a context lacking an established standard. Rather, a concordance rate of 90% was selected as a result of a consensus process after discussions with multiple technicians operating across North America. In addition, a 90% concordance rate was selected to reflect the reality that a technician manually operating an FTIR and its accompanying software would never be expected to reach a 100% concordance rate in practice due to technological limitations, sample composition and operator skill.

## 3 Results

### 3.1 Cumulative Concordance and Concordance Over Time

A total of 362 samples were collected between the two technicians over the course of their training periods, with Technician A collecting 151 samples and Technician B collecting 211 samples. The overall and technician‐specific concordance metrics are provided in Table 1. The overall cumulative concordance rate for Technician A was 90.7% (137/151) and 92.9% (196/211) for Technician B. Of Technician A's 14 discordant samples during the training period, there were 5 instances of identifying an extra component that was not present in the sample and 9 instances of missing a component that was present in the sample. Comparatively, Technician B's 15 discordant samples were comprised of 7 instances of adding an extra component that was not present in the sample and 8 instances of missing a component that was present in the sample.

**TABLE 1:** Summary of discordant and concordant samples for each technician of the New York City Health Department drug checking program.

Technician	Sample information		Total samples
	Discordant samples	Concordant samples	
Technician A	14	137	151
Technician B	15	196	211
Total samples	29	333	362

*Note:* All samples were collected between November 2021 and December 2023.

**FIGURE 1:**
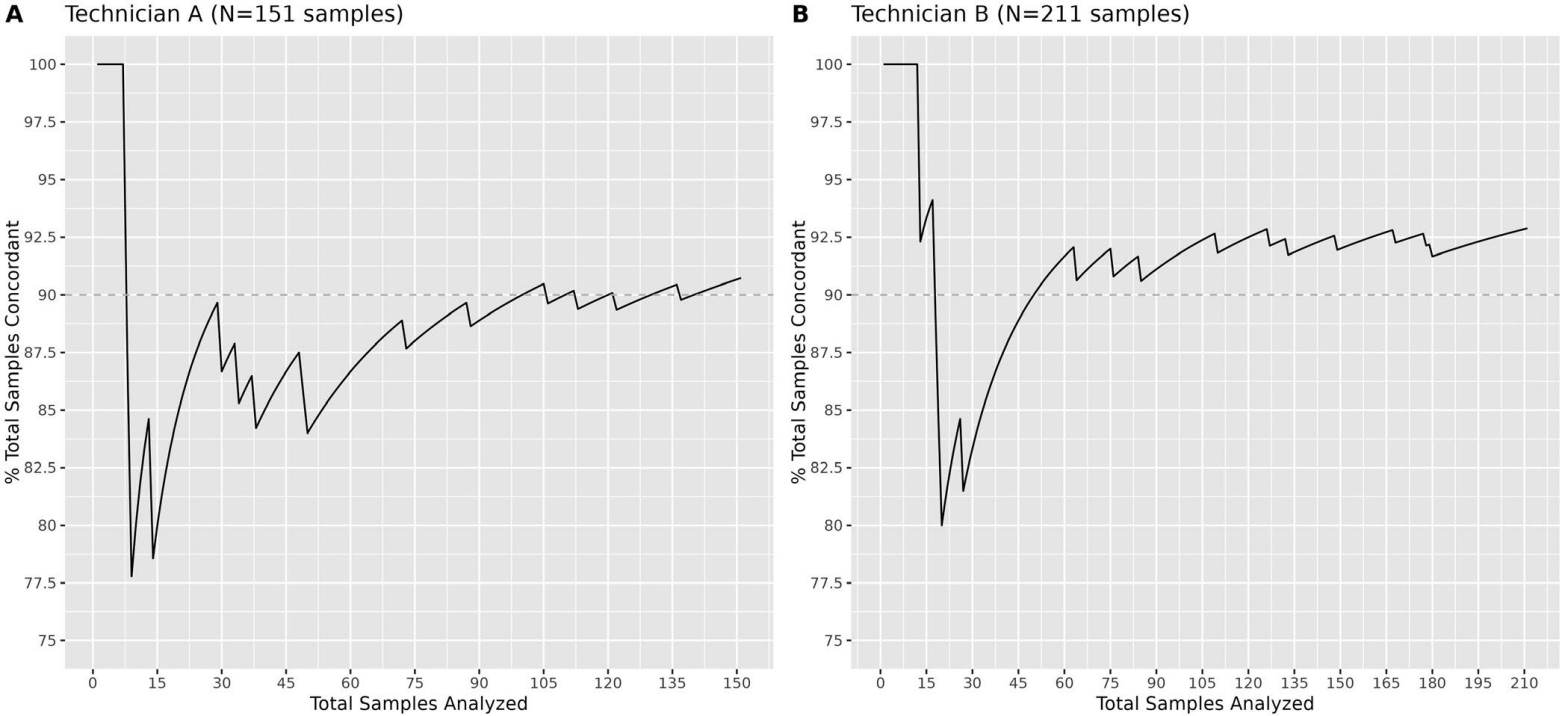
Visualisation of drug checking technician accuracy as measured by percent of total samples concordant over the total number of samples encountered. Concordance was assessed each time a new set of secondary laboratory results was received. All samples were collected as part of the New York City Health Department drug checking program between November 2021 and December 2023. (A) Technician A (*N* = 151 samples) and (B) Technician B (*N* = 211 samples).

Concordance metrics were also analysed and visualised over time (Figure 1). Technician A first reached the target measure of a cumulative 90% concordance rate on the 100th sample. Technician A then varied slightly below and slightly above the target concordance rate for the remainder of the training period, concluding their training period above the target concordance rate. Technician A's cumulative concordance rate never dropped below 77.5%. Comparatively, Technician B first reached the target concordance rate after 50 samples and, through the remainder of their training period, stayed above the target concordance rate. Technician B's lowest cumulative concordance rate was 80%. Both Technician A and Technician B improved over time as demonstrated by increases in their cumulative concordance rates as more samples were analysed The length of a technician's training period was impacted by shifts in participant engagement and the time it took to receive results back from the secondary laboratory testing partner.

### 3.2 Examples of Concordant and Discordant Samples

As previously mentioned, samples were considered concordant or discordant for different reasons. To illustrate the variety of situations encountered during the concordance evaluation process, we include several examples of sample‐level concordance evaluations in Table 2. The most common type of concordant sample was when the technician was able to identify all active compounds in a sample or all those compounds that were present in ratios such that they could have plausibly been identified using the FTIR (see, e.g., Table 2 Sample Number 1, 2, 7). Precursors such as 6‐MAM, 6‐MAC and 4‐ANPP, among others, were typically present below the detection limit, if present at all. Missing these compounds in a scan did not contribute towards a discordant evaluation. As shown in Table 2, samples were evaluated as discordant if the technician identified an extra active compound or if the technician missed an active compound that was present in a large enough ratio to be detected on the FTIR. Extra active compounds were only identified in a small proportion of the total samples (12/362 or 3%) and these extra compounds included levamisole, quinine, phenobarbital, procaine, phenacetin and xylazine.

**TABLE 2:** Examples of concordance analysis results for select samples encountered in the New York City Health Department drug checking program.

Technician	Sample reference number	Technician‐identified FTIR results	Secondary laboratory testing results	Concordance analysis evaluation	Explanation of evaluation decision
Technician A	1	Cocaine Phenacetin	Cocaine (1p) Phenacetin (0.15p)	Concordant	All active substances identified.
Technician A	2	Fentanyl Caffeine	Fentanyl (1p) Caffeine (1p) Quinine (0.1p) Para‐Fluorofentanyl (trace) Heroin (trace)	Concordant	It is not possible to identify quinine, para‐fluorofentanyl and heroin due to the detection limit of FTIR, as well as overlap of heroin and caffeine signals. All major other major active substances identified.
Technician A	3	Procaine Fentanyl	Tramadol (3.5p) Procaine (2.1p) Fentanyl (1p) Lidocaine (trace) Caffeine (trace) Acetaminophen (trace)	Concordant	At the time, tramadol was not present in the technician FTIR libraries. The technician identified all other active components that were present in large enough amounts.
Technician A	4	Methamphetamine Levamisole	Methamphetamine	Discordant	The technician identified an extra substance (levamisole) that was not present in the sample.
Technician A	5	Fentanyl	Quinine (7p) Heroin (1p) Fentanyl (1p) Caffeine (0.4p) 6‐MAM	Discordant	The technician missed heroin, quinine and caffeine.
Technician A	6	Caffeine	Heroin (1p) Caffeine (0.53p) Quinine (0.5p) Fentanyl (0.15)	Discordant	The technician missed heroin, quinine and fentanyl.
Technician B	7	Heroin Fentanyl	Heroin (1p) 6‐MAM Fentanyl (trace) Acetylcodeine 4‐ANPP Morphine Cocaine (trace)	Concordant	The technician identified active substances that met detection limit. The precursors (4‐ANPP) and impurities (acetylcodeine) were not in FTIR libraries.
Technician B	8	Heroin Fentanyl Phenacetin	Heroin (1.4p) Fentanyl (1p) Cocaine (0.18p) 4‐ANPP	Discordant	The technician identified an extra substance (phenacetin) that was not in the sample.

*Note:* All samples were collected between November 2021 and December 2023. When available, the secondary testing laboratory, CFSRE, provided parts (p) estimates for compounds identified using in‐house criteria.

Abbreviation: FTIR, Fourier transform infrared.

With respect to the additional findings that were not confirmed by the laboratory, the majority were cutting agents or common adulterants that have little to no known adverse effects with acute consumption. Reporting these in samples when they are not present is unlikely to negatively impact participants. Comparatively, samples considered discordant due to missed compounds (17/362 or 4%) included misses of heroin, quinine, caffeine, procaine, levamisole, xylazine and quetiapine. It should be noted that for some discordant results, the missed compounds have limited potential for immediate adverse events associated with their consumption (e.g., caffeine), however, chronic consumption of other compounds can lead to long term adverse effects on the body (e.g., levamisole) [22]. Of more significance are missed compounds like heroin, where consumption could lead to immediate adverse events. These misses were limited to 29/362 samples (8.0%) and generally decreased with technician competency over time. The majority of misses were associated with heroin findings not detected by the FTIR but detected in the secondary laboratory results. There are multiple possible reasons for these heroin‐specific issues, such as heroin signals being masked by common cuts like caffeine and paracetamol [23]. In instances where heroin was missed, it tended to be a lower concentration relative to other compounds (based on GC/MS area) relative to other compounds in the mixture. Further, all the heroin missed samples contained another opioid that was identified by drug checking techniques like FTIR and/or fentanyl test strips.

## 4 Discussion

Drug checking is a harm reduction practice that has continued to gain traction in the United States. When this study was executed, there were limited resources providing guidance drug checking technician training. Now, there are a growing number of materials offered by credible groups established in the field, ranging from the specific competencies required for a drug checking technician to courses providing training on the use of an FTIR for drug checking [24, 25]. In this article, we described a method that articulates a more formalised approach to training technicians via concordance analysis for the assessment of technicians operating spectroscopy‐based drug checking services. The results show that technicians with no prior experience in FTIR were able to demonstrate proficiency and successfully use the instrument as part of a harm reduction service. Technicians were able to meet our proposed definition of accuracy—90% concordance rate—in training periods that ranged from 33 to 38 weeks. Findings from this method also highlight general improvement over the course of the training period. Additional advanced training may allow technicians to reach even higher levels of concordance.

Establishing rigorous methods for the assessment of drug checking technician proficiency regarding spectroscopy‐based technologies is a difficult but important endeavour. In the context of assessing reliability in chemistry, a number of measurements and constructs have been developed over time [26]. The qualitative interpretation of spectra (i.e., different technicians looking at the same spectra may identify different sets of compounds) creates opportunities for additional research into techniques that may be useful in evaluating the consistency of information produced by drug checking technicians (e.g., inter‐rater reliability). Additionally, it may be important to assess the need for substance‐specific sensitivity and specificity as technician accuracy is especially important in the context of identifying active compounds within samples sold as opioids. Although there is an established history of unregulated drug samples containing multiple compounds, much of this evidence has been generated by advanced laboratory equipment that is difficult, cost prohibitive or unrealistic to deploy in field settings [27]. Understanding the abilities and limits of spectrometry‐based technologies that can be use in real‐world contexts remains an area of active research. A limited set of known challenges for these technologies are recognised in the drug checking field—such as signal masking, limit of detection, substances without entries in reference libraries and sample scan quality—but the shifting composition of existing drug supplies and emergence of novel psychoactive substances will pose challenges to drug checking operations [28]. Additional assessments of spectrometers on real‐world drug samples outside of concordance frameworks can help advance the understanding as to what is possible and realistic with these technologies in drug checking programs.

Our proposed training methodology did not employ a concordance structure specific to sample type because all drug checking sites were located within SSPs that served people using different types of drugs. Programs with a lower volume of certain sample types may need to structure their concordance analysis such that a set proportion of analysed samples are met before assessing technician concordance. An important but complicated aspect of the concordance analysis method described above is the use of secondary laboratory testing. Utilising a secondary laboratory for testing allows independent and unbiased comparison of technician findings to a more sophisticated reference source as well as providing additional benefits like high sensitivity to detect low level, highly potent drugs that may not be detected using techniques like FTIR. Laboratory testing may also allow for the identification of new and emerging drugs or adulterants that are not yet available in FTIR reference libraries. Additionally, partnering with a laboratory offers drug checking technicians the opportunity to communicate with a broader network of toxicologists and chemists that can aid in the interpretation of trends as well as provide context for the potential harms that may be associated with the consumption of drugs and/or drug mixtures.

It is important to note that increasing numbers of drug checking programs may consider FTIR and/or point‐of‐care FTIR as one of the many evidence‐based strategies to reduce harm among PWUD. Having robust, evidence‐based training procedures and competency protocols in place is pivotal as more technicians, who may not be formally trained in the use of analytical instrumentation, interpret spectra. The proposed methodology of concordance analysis aims to fill a gap by providing a practical competency assessment protocol. However, the successful deployment of concordance analysis will be impacted by several factors. For example, the adequate training of drug checking technicians to operate spectroscopy technologies is time‐ and resource‐intensive. Beyond the cost of acquiring a spectroscopy‐based instrument, its maintenance and subscription to relevant reference libraries [29], there is also the cost of sending drug samples for secondary laboratory testing. This is especially challenging during the technician training period if programs aim to send every sample for secondary laboratory testing. In practice, smaller programs may not be able to pay for secondary laboratory testing for every sample. In this instance, the strategic selection of samples may be necessary to preserve resources. In addition, the general paucity of technician trainers may slow the speed with which new drug checking technicians can be trained. Maintaining high quality and consistent training regimens is also important to avoid differences in the quality of training across programs. Strong, collaborative relationships between the secondary laboratory partner and drug checking technicians can help technicians to better understand the findings they generate from point‐of‐care technologies. If possible, programs should account for these costs and factors accordingly as concordance analysis is an important part of the technician training process and helps ensure that technicians provide accurate information to PWUD.

Evolving drug checking procedures raise important questions as to how variations in technology and software may impact drug checking results and related outputs, including concordance evaluations. Drug checking programs use a variety of spectroscopy‐based technologies, each of which may be produced by different companies and accompanied by bespoke software requiring updates over time. The instruments themselves require maintenance and close care to ensure that scans taken from samples are not degraded in quality. Moreover, different versions of spectral libraries used by the accompanying software are updated at different increments but may not be universally adopted across all drug checking programs at the same time. Each of these steps in current drug checking procedures is a source of variation that can impact drug checking results and concordance. While some of these sources of variation can be controlled through standardised operating procedures, innate technological limitations may persist. Identifying these sources of variation, understanding the limits of drug checking technologies and employing risk mitigation strategies can help promote a rigorous and shared understanding as to what drug checking results mean in practice.

Several factors enabled success for the concordance proof‐of‐concept methodology and technician training period. The drug checking program received institutional support and funding from a large public health agency over a relatively long period, as well as active participation by the community‐based organisation hosting the drug checking program within their suite of existing services. On an individual level, the drug checking technicians undergoing training were people with lived experience, had a strong curiosity about drug culture and use, demonstrated intermediate computer proficiency and had prior employment experience working in behavioural health settings. During the training phase those in training and the trainers met regularly to review drug checking results and address recurring issues. Drug checking results and concordance determinations were stored digitally in a single place, enabling a strong sense of organisation and growth as the training progressed. These factors place an emphasis on individual skills and experience, which may help expand the pool of people who can be considered for this work. These enabling factors and successful outcomes of the training program may support emerging work that underscores the benefits of hiring people with a lived experience involving substance use in the health workforce [30, 31].

As has been recognised elsewhere, drug checking technicians are increasingly expected to both successfully operate sophisticated equipment and provide accompanying education, connections to care, and more [3]. While the present research proposes a method to assess the former, we do not offer a methodology for assessing the latter. Demonstrating competency in both skillsets is essential to provide a high‐quality service to PWUD. In addition, recent work has highlighted a number of barriers and facilitators to implementing drug checking services that are separate from technician competency [29]. For example, Carroll et al. identified that the implementation of spectroscopy‐based drug checking services was facilitated by, ‘…[a] shared culture of harm reduction at the SSP (which in turn fostered shared implementation goals and beliefs about the intervention among staff persons), horizontal organizational structures, staff identification with the SSP, strong relationships with external funders and peer organizations tackling the same challenges, and the ongoing integration of lessons learned to the implementation strategy over time’ [29]. These factors are especially important to consider as drug checking plays an active role in the creation of knowledge around drug use [32]. Expansive ethnographic work by Betsos et al. found that ‘…there are multiple types of knowledge required to construct and interpret drug‐checking results for both PWUD and drug‐checking technicians’, ranging from personal information and experience provided by participants to scientific information rendered by different drug checking technologies [32]. Furthermore, Betsos et al. highlighted that SSP participant curiosity played a strong role in the decision to test substances and that drug checking conducted by peers created an important opportunity for the trans‐production of knowledge for all those involved [32]. For example, Betsos et al. provide an example as to how these multiple knowledges come into play in FTIR detection of inactive, cut and buffer substances, wherein the drug checking technician identifies the substance and the person providing the sample (i.e., a person with lived experience) provides possible explanations as to why other substances, in this case caffeine and mannitol, might be present. Similar observations on knowledge co‐production appear in Estrada et al. [18]. In this work, a limited number of drug samples submitted to a drug checking program produced unexpected drug checking results (e.g., a sample sold as cocaine producing a positive fentanyl test strip result); it was only through conversations with the people who had submitted the drug samples that unexpected results could be explained [18]. Future qualitative research could explore how the multiple, sometimes overlapping actors involved in drug checking services—people submitting samples for drug checking, the drug checking staff, host organisation and information consumers—ultimately co‐produce knowledge about the unregulated drug supply.

### 4.1 Limitations

The proposed method of concordance analysis has a few limitations worth noting. First, concordance analysis ultimately classifies whether technicians are trained based on a cumulative measure that has no reference standard and is not based on an established definition. There are potential issues with this aspect of the proposed method. The selection of a target measure for concordance analysis may be subject to Goodhart's Law, wherein unintended consequences arise due to the pressure to meet a certain standard. If the target measure of concordance is too high, it may be difficult for even well‐trained technicians to consistently operate at near‐perfect accuracy. If the target measure is too low, the quality of information provided to PWUD declines. It is important that accurate information is provided to people using drug checking services in every instance. This is particularly important as poor‐quality information can have extremely high consequences in certain situations—for example, a drug checking technician missing the presence of opioids in a sample thought to be a non‐opioid, especially for an opioid‐naïve individual intending to consume the substance. More broadly, the lack of an established definition about what makes a sample concordant is a related and larger limitation that could undermine the calculated concordance rates presented here. The definition we propose here may be too broad, thus resulting in artificially high concordance rates. Future research should consider different definitions of concordance to better understand the impact of definitions on estimated technician performance. For example, alternative definitions may more closely leverage information from the instruments used in secondary laboratory testing in concordance determinations. Such critical re‐assessments would help to add understanding as to the interpretability of concordance rates and help identify what target measure comports with the needs of community‐based programs offering point‐of‐care drug checking.

Second, the proposed method of concordance does not consider substance‐specific sensitivity and specificity, which has been considered elsewhere, nor variation in concordance between different drug classes [10]. The concordance metrics presented here are meant to describe the technician's ability to identify all active compounds present and observable in a sample given the instrument's limits. This ability is conceivably impacted by other factors, such as the quality and speed of feedback provided by the technician trainer, the turnaround of secondary laboratory results to rectify error patterns and the variety of samples submitted to the drug checking service. Furthermore, it is conceivable that technician performance (and concordance metrics) could be driven by the *type* of samples that the drug checking program receives, ultimately resulting in technician skill that varies by substance class. For example, in New York City, stimulant samples tend to contain a limited number of compounds, all of which are readily identifiable via FTIR. In comparison, opiate samples tend to contain a higher number of compounds that can be challenging to analyse even for a trained drug checking technician. Programs that do not receive a variety of samples may have less practice analysing samples of a certain kind, resulting in information of differing quality. Future research should leverage the growing community of drug checking technicians across the United States and globally to reduce the impact of these limitations on the delivery of high‐quality information to PWUD.

Third, the practical method described here focuses on concordance during a technician's training period. Once the target accuracy of 90% was reached, the technicians‐in‐training were considered trained and the ongoing concordance evaluation was stopped. It is plausible that a technician's concordance rate continued to exhibit variation beyond the samples included in the training period. Moreover, externalities like changes in the common cuts found in the unregulated drug supply, samples with a higher number of unique substances in varying concentrations or a change in the sample type routinely encountered by technicians could all impact a technician's accuracy when using FTIR to identify compounds in drug samples. Longstanding programs providing spectroscopy‐based drug checking may be able to conduct concordance analysis over a longer timeframe to provide insight into whether and how technician accuracy may vary in the post‐training period.

Fourth, the results presented here should be interpreted in consideration of a small technician sample size and a single geographic context. The evaluation of the practical methodology included only two people in the training pool and both were trained within a single geographic area. Concordance results and training progression may not generalise to different training procedures or other jurisdictions. At present, it is difficult to determine whether the levels of concordance observed here are realistically replicable given significant variation in program structure, capacity and observed drug supplies. Future research could include a larger number of technicians across multiple jurisdictions to gain a better understanding of technician performance and the time required to achieve different levels of proficiency. For example, cross‐sectional analysis of technicians with different skill levels and for smaller amounts of time may provide valuable insight into how a technician's concordance rate changes with experience.

## 5 Conclusion

Concordance analysis offers a practical method of accuracy assessment for individuals operating advanced instruments in the provision of drug checking as a harm reduction service. Concordance analysis is an ongoing quality assurance process that can be carried out to continuously improve the quality of information provided by drug checking technicians to PWUD. Essential components of concordance analysis are a trained instrument operator and a secondary laboratory testing partner. Results from our method demonstrate that individuals with no prior training in FTIR operation could reliably identify multiple compounds present in drug samples. This methodology may prove useful to drug‐checking technician trainers and other drug‐checking programs offering technician‐operated FTIR.

## Author Contributions

Y.E. and L.G. guided the analysis, provided review of quantitative results and contributed original writing. Y.E. managed the scientific program and reviewed the analysis. J.S. provided quantitative analysis and original writing. M.B., S.S. and A.M. helped guide the analysis, provided essential chemical analysis services, contributed original writing and reviewed the written report.

## Funding

This project was supported by the Centers for Disease Control and Prevention (CDC) of the U.S. Department of Health and Human Services (HHS) as part of Overdose Data to Action (CDC‐RFA‐CE19‐1904) and Overdose Data to Action: LOCAL (CDC‐RFA‐CE‐23‐0003). The contents are those of the author(s) and do not necessarily represent the official views of, nor an endorsement by, CDC/HHS or the US Government.

## Ethics Statement

The study was submitted to and approved by the New York City Department of Health and Mental Hygiene Institutional Review Board as a research activity (protocol #21‐034).

## Conflicts of Interest

The authors declare no conflicts of interest.

## Data Availability

The data that support the findings of this study are available from the corresponding author upon reasonable request.

## References

[1] Trans European Drug Information , TEDI Guidelines: Drug Checking Methodology. 1st ed. March 31st, 2022. (Editions for social change, 2022), https://tedinetwork.org/wp‐content/uploads/2023/10/TEDI_Guidelines_A5.pdf.

[2] J. N. Park , J. Tardif , E. Thompson , J. G. Rosen , J. A. S. Lira , and T. C. Green , “A Survey of North American Drug Checking Services Operating in 2022,” International Journal on Drug Policy 121 (2023): 104206. 37797571 10.1016/j.drugpo.2023.104206 PMC10843152

[3] L. Gozdzialski , B. Wallace , and D. Hore , “Point‐Of‐Care Community Drug Checking Technologies: An Insider Look at the Scientific Principles and Practical Considerations,” Harm Reduction Journal 20, no. 1 (2023): 39. 36966319 10.1186/s12954-023-00764-3 PMC10039693

[4] J. C. Halifax , L. Lim , D. Ciccarone , and K. L. Lynch , “Testing the Test Strips: Laboratory Performance of Fentanyl Test Strips,” Harm Reduction Journal 21, no. 1 (2024): 14. 38238757 10.1186/s12954-023-00921-8 PMC10795297

[5] S. E. Rodriguez‐Cruz , “Evaluating the Sensitivity, Stability, and Cross‐Reactivity of Commercial Fentanyl Immunoassay Test Strips,” Journal of Forensic Sciences 68, no. 5 (2023): 1555–1569. 37420315 10.1111/1556-4029.15332

[6] M. Ramsay , L. Gozdzialski , A. Larnder , B. Wallace , and D. Hore , “Fentanyl Quantification Using Portable Infrared Absorption Spectroscopy. A Framework for Community Drug Checking,” Vibrational Spectroscopy 114 (2021): 103243.

[7] F. Giulini , E. Keenan , N. Killeen , and J. H. Ivers , “A Systematized Review of Drug‐Checking and Related Considerations for Implementation as A Harm Reduction Intervention,” Journal of Psychoactive Drugs 55, no. 1 (2023): 85–93. 35060837 10.1080/02791072.2022.2028203

[8] L. Gozdzialski , A. Hutchison , B. Wallace , C. Gill , and D. Hore , “Toward Automated Infrared Spectral Analysis in Community Drug Checking,” Drug Testing and Analysis 16, no. 1 (2024): 83–92. 37248686 10.1002/dta.3520

[9] R. Goncalves , K. Titier , V. Latour , et al., “Suitability of Infrared Spectroscopy for Drug Checking in Harm Reduction Centres,” International Journal on Drug Policy 88 (2021): 103037. 33207305 10.1016/j.drugpo.2020.103037

[10] T. C. Green , J. N. Park , M. Gilbert , et al., “An Assessment of the Limits of Detection, Sensitivity and Specificity of Three Devices for Public Health‐Based Drug Checking of Fentanyl in Street‐Acquired Samples,” International Journal on Drug Policy 77 (2020): 102661. 31951925 10.1016/j.drugpo.2020.102661

[11] K. W. Tupper , K. McCrae , I. Garber , M. Lysyshyn , and E. Wood , “Initial Results of a Drug Checking Pilot Program to Detect Fentanyl Adulteration in a Canadian Setting,” Drug and Alcohol Dependence 190 (2018): 242–245. 30064061 10.1016/j.drugalcdep.2018.06.020

[12] M. K. Laing , L. Ti , A. Marmel , et al., “An Outbreak of Novel Psychoactive Substance Benzodiazepines in the Unregulated Drug Supply: Preliminary Results From a Community Drug Checking Program Using Point‐Of‐Care and Confirmatory Methods,” International Journal on Drug Policy 93 (2021): 103169. 33627302 10.1016/j.drugpo.2021.103169

[13] K. D. Wagner , P. Fiuty , K. Page , et al., “Prevalence of Fentanyl in Methamphetamine and Cocaine Samples Collected by Community‐Based Drug Checking Services,” Drug and Alcohol Dependence 252 (2023): 110985. 37826988 10.1016/j.drugalcdep.2023.110985 PMC10688611

[14] British Columbia Centre on Substance Use (BCCSU) , Drug Checking Operational Technician Manual Version 2 (Providence Health Care (PHC), 2022).

[15] C. Schmidt and Y. Estrada , Setting up a Drug‐Checking Program: A Comprehensive Guide to Implementation (Department of Health and Mental Hygiene's (NYC Health Department), 2024), 1–60.

[16] Academy Standard Board (ASB) , Standard Practices for Proficiency Testing for Forensic Toxicology Laboratories and Breath Alchol Programs (Academy Standard Board (ASB), 2023), 1–11.

[17] ANSI National Accreditation Board , Accreditation Requirements for Forensic Testing and Calibration (ANSI National Accreditation Board, 2023), 1–20.

[18] Y. Estrada , J. Sauer , L. Dominguez , et al., “The Prevalence of Fentanyl in New York City's Unregulated Drug Supply as Measured Through Drug Checking Offered at Syringe Service Programs,” Drug and Alcohol Dependence 268 (2025): 112578. 39914191 10.1016/j.drugalcdep.2025.112578

[19] St. George's University of London , TICTAC Spectral Libraries for Drugs and New Psychoactive Substances/Legal Highs (TICTAC Communications Ltd, 2024).

[20] The BC Centre on Substance Use (BCCSU) , BCCSU FTIR‐ATR Library (BC Centre on Substance Use (BCCSU), 2026).

[21] Scientific Working Group for the Analysis of Seized Drugs (SWGDRUG) , SWGDRUG Infrared Spectral Library (Scientific Working Group for the Analysis of Seized Drugs (SWGDRUG), 2024), https://www.swgdrug.org/ir.htm.

[22] K. Midthun , A. Mohr , T. Browne , D. Martin , and B. Logan , Levamisole: A Toxic Adulterant Found in Illicit Street Drugs (Center for Forensic Science Research & Education (CFSRE), 2020).

[23] S. F. Williams , R. Stokes , P. L. Tang , and A. M. Blanco‐Rodriguez , “Detection & Identification of Hazardous Narcotics and New Psychoactive Substances Using Fourier Transform Infrared Spectroscopy (FTIR),” Analytical Methods 15, no. 26 (2023): 3225–3232. 37341678 10.1039/d3ay00766a

[24] BC Centre on Substance Use (BCCSU) , “FTIR Training, Drug Checking Courses,” accessed July, 20, https://drugcheckingbc.ca/training/.

[25] Drug Checking for the People , Defining Practice: Competencies for Drug Checking Technicians (Remedy Alliance, 2026).

[26] J. Harshman and E. Yezierski , “Test–Retest Reliability of the Adaptive Chemistry Assessment Survey for Teachers: Measurement Error and Alternatives to Correlation,” Journal of Chemical Education 93, no. 2 (2015): 239–247.

[27] C. Cole , L. Jones , J. McVeigh , A. Kicman , Q. Syed , and M. Bellis , Cut: A Guide to Adulterants, Bulking Agents and Other Contaminants Found in Illegal Drugs (Faculty of Health and Applied Social Sciences, Liverpool John Moores University, 2010).

[28] P. Gonzalez‐Nieto , B. Wallace , C. Kielty , et al., “Not Just Fentanyl: Understanding the Complexities of the Unregulated Opioid Supply Through Results From a Drug Checking Service in British Columbia, Canada,” International Journal on Drug Policy 138 (2025): 104751. 40022963 10.1016/j.drugpo.2025.104751

[29] J. J. Carroll , S. Mackin , C. Schmidt , M. McKenzie , and T. C. Green , “The Bronze Age of Drug Checking: Barriers and Facilitators to Implementing Advanced Drug Checking Amidst Police Violence and COVID‐19,” Harm Reduction Journal 19, no. 1 (2022): 9. 35120531 10.1186/s12954-022-00590-z PMC8814788

[30] K. Closson , R. McNeil , P. McDougall , et al., “Meaningful Engagement of People Living With HIV Who Use Drugs: Methodology for the Design of a Peer Research Associate (PRA) Hiring Model,” Harm Reduction Journal 13, no. 1 (2016): 26. 27717364 10.1186/s12954-016-0116-z PMC5054577

[31] C. A. Gagne , W. L. Finch , K. J. Myrick , and L. M. Davis , “Peer Workers in the Behavioral and Integrated Health Workforce: Opportunities and Future Directions,” American Journal of Preventive Medicine 54, no. 6 Suppl 3 (2018): S258–S266. 29779550 10.1016/j.amepre.2018.03.010

[32] A. Betsos , J. Valleriani , J. Boyd , and R. McNeil , “Beyond Co‐Production: The Construction of Drug Checking Knowledge in a Canadian Supervised Injection Facility,” Social Science & Medicine 314 (2022): 115229. 36274456 10.1016/j.socscimed.2022.115229

